# Measles Virus Genotypes Causing Outbreaks in Tanzania, 2022–2024

**DOI:** 10.3390/v18020182

**Published:** 2026-01-29

**Authors:** Fausta S. Michael, Maria E. Kelly, Lawrence A. Mapunda, Monica F. Francis, Naimi H. Mbogo, Azizi H. Ituka, Kelvin A. Tenga, Ambele E. Mwafulango, Mariam M. Mirambo, Stephen E. Mshana, Gerald Misinzo

**Affiliations:** 1 Immunization and Vaccine Development (IVD) Programme, Ministry of Health, Dodoma P.O. Box 743, Tanzania; fausta.selemani@afya.go.tz; 2 World Health Organization, Dar es Salaam P.O. Box 9292, Tanzania; kellyma@who.int (M.E.K.); nmmazzuki@gmail.com (N.H.M.); 3 National Public Health Laboratory, Ministry of Health, Dar es Salaam P.O. Box 9083, Tanzania; lawrence.mapunda@afya.go.tz (L.A.M.); francismonica6@gmail.com (M.F.F.); azizi.ituka@afya.go.tz (A.H.I.); kelvin.tenga@afya.go.tz (K.A.T.); ambele.mwafulango@afya.go.tz (A.E.M.); 4 Department of Microbiology and Immunology, Weill Bugando School of Medicine, Catholic University of Health and Allied Sciences, Mwanza P.O. Box 1464, Tanzania; mmmirambo@bugando.ac.tz; 5 SACIDS Foundation for One Health, Sokoine University of Agriculture, Morogoro P.O. Box 3000, Tanzania; gerald.misinzo@sacids.org

**Keywords:** measles virus, genotypes, nucleoprotein gene, Tanzania

## Abstract

Globally, 24 measles virus genotypes have been detected, and these genotypes have been classified into eight clades based on 450 nucleotides of the C-terminal region of the nucleoprotein gene. Genotype B3 is predominant in Africa, but there are limited data from Tanzania since the introduction of the second dose of measles-containing vaccine in 2014. A total of 129 nasopharyngeal samples and corresponding sera were collected during measles outbreaks between 2022 and 2024. Viral RNA was extracted from nasopharyngeal swabs prior to RT-qPCR and sequencing of a 450-nucleotide segment of the nucleoprotein (N) gene. Out of 129 nasopharyngeal samples, 73 (56%) were successfully amplified and identified as endemic measles virus genotype B3. Nine distinct sequence identifiers were detected, with seven reported for the first time in the MeaNS database. All the Tanzanian B3 sequences were closely related and clustered with genotype B3, similar to those reported from Kenya, Ethiopia, Rwanda, Uganda, Burundi, and South Africa. On multivariate analysis, only inpatient admission status (*p* = 0.014) and positive measles IgM (*p* = 0.003) were found to be associated with positive measles RT-qPCR. Our results indicate that genotype B3 remains endemic in Tanzania and is closely related to other genotype B3 reported globally, indicating its high stability and transmissibility.

## 1. Introduction

Measles virus (MeV) is highly contagious, causing a respiratory disease that is transmitted through respiratory droplets when a person with active infection talks, coughs, or sneezes [1]. MeV RNA can be detected by RT-PCR in urine, throat swabs, and blood samples up to 14 days after rash onset, with lower detection rates 5–7 days after rash [2]. Genotypic surveillance of MeV is based on the nucleotide sequence encoding the 150 carboxyl-terminal amino acids of the nucleoprotein (N450), a region with high variability in the genome [3,4]. The World Health Organization (WHO) recognizes eight clades of MeV: A, B, C, D, E, F, G, and H. Within these clades, there are 24 recognized genotypes, designated A, B1, B2, B3, C1, C2, D1, D2, D3, D4, D5, D6, D7, D8, D9, D10, E, F, G1, G2, G3, H1, H2, and one provisional genotype, D11 [5,6,7].

Despite the existence of multiple genotypes, there is a high similarity in surface antigens among all MeV strains [3,4,8]. Therefore, all MeV isolates are classified as a single serotype, and infection or vaccination generally provides lifelong immunity. Molecular characterization has been pivotal in tracking viral genotypes over time in specific regions, enabling the differentiation between natural infections and adverse events induced by vaccination [9,10,11]. In combination with standard epidemiologic information, genotyping can be used to track transmission pathways and provide evidence for verification of measles elimination [6].

Globally, the diversity of circulating MeV genotypes is decreasing [4,5], with eighteen genotypes identified in 2003 [12]. Between 2005 and 2014, 13 genotypes were reported globally [4,13]. Between 2018 and 2024, only five genotypes (B3, D4, D8, D9, and H1) from three clades have been reported to the WHO Global Measles Nucleotide Surveillance (MeaNS) database [14]. Since 2021, MeV circulation has been linked to B3 and D8 genotypes, accounting for 97% of the genotypes reported to the MeaNS database [14,15]. This trend highlights the success of measles elimination strategies in interruption of the transmission of several genotypes [12].

Some MeV genotypes have been found to correlate with specific geographical regions: clade B is prevalent in Sub-Saharan and Central Africa, clade G in Southeast Asia, and clade H in Southeast Asia and China. Clade D viruses are more broadly distributed and endemic to Eastern Africa, parts of Europe, and the Indian sub-continent [16]. Measles clade B genotypes (B1, B2, and B3) are endemic in Sub-Saharan Africa [9,17] and have been associated with frequent importations into different parts of the world [16]; these genotypes have been detected in all six WHO regions [18]. Genotype B3, which was first detected in 1993 in Gambia, has been associated with outbreaks [19,20,21] and has been reported to be significantly more transmissible than other genotypes [17]. Genotypes D2, D4, and D10 were frequently detected in the southern and eastern parts of the African continent [22,23,24], though more recent outbreaks in Kenya, Uganda, Burundi, and Tanzania have been caused by genotype B3 [25]. Genotypes D4 and D10, which had been circulating in eastern Africa, have not been detected in the past 3 years, and D10 has not been detected anywhere since 2005 [26].

Virologic surveillance in the WHO African Region has significantly advanced, with reports of viral genotype data collected from 21 countries between 2007 and 2024 [9]. The predominant genotype was B3, accounting for 197 out of 220 sequences reported to the MeaNS database. In some African countries, genotype B2 viruses were also detected between 2002 and 2010 [27,28,29]. Genotype B3 sub-cluster B3-1 has previously been isolated from Cameroon, Ghana, Nigeria, Kenya, and Tanzania, suggesting that genotype B3-1 viruses are widely distributed throughout Africa [6,23,30,31]. The circulation of genotype B3-2 appears to be more limited to Western Africa [27]. Over the past two decades, B3, D3, and D8 MeV genotypes have been circulating in 10 West African countries, with genotype B3-1 currently dominating, especially in Nigeria, indicating endemic transmission [32].

The WHO recommends implementing virological surveillance for measles to identify indigenous strains [33]; this identification is crucial for understanding transmission patterns, assessing progress toward elimination, and monitoring immunization programs [31]. Verification of measles elimination requires documentation of at least 12 months without endemic MeV circulation, supported by an effective surveillance system [14].

Tanzania has experienced measles outbreaks of varying magnitude over the years, particularly between 1999 and 2022 [34,35,36,37,38]. Between 2018 and 2022, the country documented an increase in the number of measles cases and widespread measles outbreaks [38]. Limited data are available regarding MeV genotypes in Tanzania, representing a significant gap in monitoring progress towards measles elimination in the country. Measles genotype B3 was detected from four samples of nasal swabs, of which three had similar sequences collected from the initial cluster of laboratory-confirmed measles cases from the Muheza district in the Tanga region during a large outbreak in 2006 [36]. Since the introduction of the second dose of measles–rubella (MR2) vaccine into routine immunization in 2014, there has been no documentation of circulating measles genotypes in Tanzania. This study provides information on the circulating measles genotypes in Tanzania during the 10 years of implementing a two-dose MR vaccination policy (MR1 at 9 months and MR2 at 18 months) and provides critical evidence for assessing progress toward measles elimination by 2030 in line with the Immunization Agenda 2030 [39].

## 2. Materials and Methods

### 2.1. Study Design

This was a cross-sectional study using the data collected for routine measles surveillance involving suspected measles cases from all 195 councils who presented with rash and fever within 5 days of the onset of these symptoms. Nasopharyngeal swabs and respective serum samples were collected and shipped to the National Public Health Laboratory (NPHL) for analysis to identify the specific type of MeV genotypes causing outbreaks in Tanzania.

### 2.2. Study Population and Clinical Samples

A total of 129 non-repetitive serum samples and corresponding nasopharyngeal swabs from suspected measles cases presenting with fever and rash within 5 days of onset of the symptoms were collected according to measles and rubella surveillance guidelines for AFRO countries. The majority of the samples (127) were collected during measles outbreaks that occurred between 2022 and 2024, one sample from the measles outbreak that occurred in 2011 in Iringa, and one from the outbreak in refugees camp in Kigoma in 2020. Clinical and demographic characteristics, including age, date of symptom (rash and fever) onset, vaccination status, admission history, and other additional information were collected using the measles generic case investigation form (CIF). Sera and nasopharyngeal swabs were transported to the NPHL under a maintained cold chain. Nasopharyngeal swabs were carefully preserved in viral transport medium (VTM) throughout the collection process. Samples were initially stored at 2–8 °C for up to 7 days before testing, and, subsequently, they were maintained at −20 °C before undergoing further analyses.

### 2.3. Detection of Measles Immunoglobulin M (IgM) Antibodies

Anti-measles IgM antibodies from patient serum samples were detected using Neuroimmune EIA kits (EUROIMMUN Medizinische Labordiagnostika AG, Lübeck, Germany) following the manufacturer’s instructions and the WHO manual for the laboratory-based surveillance of measles, rubella, and congenital rubella syndrome [40].

### 2.4. RNA Extraction, Reverse-Transcription Quantitative Real-Time PCR (RT-qPCR) Detection, and Reverse-Transcription Real-Time PCR (RT-PCR) Amplification of the MeV N450 Region

Viral RNA was extracted from 129 nasopharyngeal swabs using the QIA amp Viral RNA Mini Kit (Qiagen, Hilden, Germany), according to the manufacturer’s instructions. Initial screening for MeV was performed using a US CDC-recommended RT-qPCR assay targeting the MeV nucleoprotein (N) gene, with human RNase P mRNA included as an internal control to assess RNA extraction quality and integrity. Briefly, the RT-qPCR was conducted using the forward primer (MVN1139-F): 5′-TGGCATCTGAACTCGGTATCAC-3′, the reverse primer (MVN1213-R): 5′-TGTCCTCAGTAGTATGCATTGCAA-3′, and probe (MVNP1163-P): 5′-FAM-CCGAGGATGCAAGGCTTGTTTCAGA-BHQ-3′ on an ABI 7500 Fast Real-time PCR thermocycler (Applied Biosystems, Foster City, CA, USA). The RT-qPCR reaction was performed in a final volume of 25 μL using the SuperScript III Platinum One-Step qRT-PCR Kit (Life Technologies, Carlsbad, CA, USA), according to the manufacturer’s instructions and the US CDC protocol [41,42]. The cycling conditions included a reverse-transcription step at 48 °C for 30 min, followed by enzyme activation at 95 °C for 5 min, 40 cycles of denaturation at 95 °C for 15 s, and annealing/extension at 60 °C for 1 min.

Samples yielding positive RT-qPCR results were subsequently subjected to conventional one-step RT-PCR for amplification of the MeV N450 region for molecular characterization and genotyping. Briefly, RT-PCR was conducted using primers MeV-216 (5′-TGGAGCTATGCCATGGGAGT-3′) and MeV-214 (5′-TAACAATGATGGAGGGTAGG-3′), according to the manufacturer’s instructions and the US CDC protocol [41,42]. The Qiagen One-step RT-PCR kit (Qiagen, Hilden, Germany) was used to amplify the desired gene fragment. The RT-PCR mixture contained 27.5 μL of nuclease-free water, 10 μL of 5X Qiagen One Step buffer, 2 μL of dNTP, 1.5 μL of 20 µM MeV214 primer, 1.5 μL of 20 µM MeV216 primer, 0.5 μL of RNase inhibitor, 1 μL of Qiagen enzyme, and 5 μL of the RNA. The RT-PCR process was conducted in a 96-well conventional GeneAmp 9700nPCR system thermocyclers (Applied Biosystems, Foster City, CA, USA). The cycling conditions included reverse transcription for 30 min at 50 °C, initial denaturation for 15 min at 95 °C, followed by 40 cycles of denaturation at 95 °C for 30 s, annealing at 55 °C for 30 s, and extension at 72 °C for 30 s, with a final extension at 72 °C for 10 min and storage at 4 °C. Subsequently, the RT-PCR products were visualized on a 1% agarose gel after electrophoresis for 1 h at 100 V.

### 2.5. Measles Genotyping

The amplified N450 fragments were purified using ExoSAP-IT PCR product cleanup reagent (Applied Biosystems, Foster City, CA, USA). Purified PCR products were sequenced using Sanger sequencing with primers MeV214 and MeV216, as specified in the US CDC protocol [41,42]. The Big-Dye terminator V3.1 ready reaction cycle sequencing kit (Applied Biosystems, Foster City, CA, USA) was used during sequencing PCR in accordance with the manufacturer’s guidelines on GeneAmp 9700 PCR system thermocyclers (Applied Biosystems, Foster City, CA, USA). Finally, the purification of the cycle sequencing product was achieved using the BigDye XTerminator purification kit (Applied Biosystems, Foster City, CA, USA), and nucleotide sequencing was performed on a 3500 XL genetic analyzer (Applied Biosystems, Foster City, CA, USA). Sequence analysis was carried out using the web-based RECall v2.32.1 software (https://recall.bccfe.ca/updates, accessed on 10 December 2025). The 450-long nucleotide fragments derived from RECall v2.32.1 were analyzed for the closest match against 28 reference nucleotide sequences and, subsequently, against eight additional genotype B3 sequences from GenBank, using the Clustal W algorithm implemented in MEGA 11 [9]. All nucleotide sequences generated in this study adhered to the WHO nomenclature and were submitted to the Measles Nucleotide Surveillance (MeaNS) database and NCBI GenBank with accession numbers: PX854700-PX854771 (BankIt3041377).

### 2.6. Data Analysis and Phylogenetic Analysis

Descriptive data analysis was performed using STATA software version 15 (StataCorp, College Station, TX, USA). Continuous independent variables such as age and days from rash/fever onset to sample collection (days–onset) were summarized with median and interquartile range (IQR), while categorical independent variables (sex, vaccination status, admission status, clinical symptoms, and age categories of <5 years, 5–9.99 years, and ≥10 years) were summarized as proportions. The two-sample Wilcoxon’s rank-sum (Mann–Whitney) test was used to compare median duration since vaccination to sample collection among those that received either a single dose or two doses in relation to laboratory-confirmed measles cases. Chi-squared test was used in univariate and multivariate logistic regression analysis to determine factors associated with RT-PCR positivity (dependent variable). All factors with a *p*-value < 0.05 in the univariate analysis were included in the multivariate model, which was adjusted for age and sex. A *p* value of <0.05 was considered statistically significant. The odds ratios (ORs) with respective 95% Confidence Intervals (CIs) and *p*-values were presented.

Phylogenetic trees were constructed in MEGA 11 using the maximum parsimony method with MeV B3 genotypes outside Africa as the outgroup and 1000 bootstrap replicates. Clade, genotype, and cluster attributions were established by assessing the formed clusters between the analyzed sequences and reference sequences of known clades [9].

### 2.7. Ethical Considerations

This study received ethical approval to use the data and archived samples collected between 2022 and 2024 from the Joint CUHAS/BMC Research Ethics and Review Committee (CREC/953/2025), and permission was granted from the Ministry of Health. The collection of the data adhered to the WHO protocol for measles and rubella surveillance (https://www.afro.who.int/sites/default/files/2017-06/who-african-regional-measles-and-rubella-surveillance-guidelines_updated-draft-version-april-2015_1.pdf) (accessed on 22 January 2026).

## 3. Results

### 3.1. Demographic Characteristics of Measles Cases

Table 1 below summarizes the sociodemographic and clinical characteristics of 129 participants from whom the serum samples and corresponding nasopharyngeal swabs were collected between 2022 and 2024. All participants presented with fever and rash, and the majority also had cough 109 (84.5%) and runny nose 93 (72.1%). The median age was 60 (IQR: 24–109) months. There was a slight male predominance, 71 (55%), and 52 (40.3%) samples were from participants who were not vaccinated against measles (zero dose).

Regarding those who received only the first dose (*n* = 13, MR1), the median duration between vaccination and sample collection was 36 months, IQR (7–124), while among those that received two doses (*n* = 24), the median duration between last vaccination (MR2) and sample collection was 21 months, IQR (11–60). No significant difference (*p* = 0.831) was observed regarding median duration since vaccination among negative cases (*n* = 30, 25.5 months, IQR: 9–66) and laboratory-confirmed cases—either measles IgM positive or RT-qPCR positive (*n* = 7, 21 months, IQR: 2–124). In addition, a total of 36 (27.9%) suspected cases were admitted, with no case of neurological complications of measles reported during hospitalization.

### 3.2. Factors Associated with RT-qPCR Positivity

Measles-specific IgM antibodies were detected in 78 (60.4%, 95% CI: 51.9–68.8) of the 129 serum samples tested. Of the 129 nasopharyngeal samples tested using measles RT-qPCR, 73 (56.5%, 95% CI: 47.9–65.0) were found to be positive. A total of 58 (44.9%) participants were positive for both measles-specific IgM and measles RT-qPCR. Out of 51 participants who were measles-specific IgM negative, 15 (29.4%) were measles RT-qPCR positive. In addition, of 78 participants who were measles-specific IgM positive, 20 (25.6%) were RT-qPCR negative. The median duration from onset of symptoms to sample collection among those who were measles IgM positive but RT-qPCR negative (*n* = 20) was 3 days, IQR: 1–4.5; for those who were measles IgM negative but RT-qPCR positive (*n* = 15), it was 3 days, IQR: 2–4; and for those who were positive for both (*n* = 58), it was 3 days, IQR: 2–4.5.

On multivariate analysis, only inpatient admission status (OR: 4.181, 95% CI: 1.332–13.123, *p* = 0.014) and positive measles IgM (OR: 5.013, 95% CI: 1.726–14.561, *p* = 0.003) were significantly associated with positive RT-qPCR (Table 2). Further analysis revealed that, out of 36 inpatients, 32 (88.9%) were either not vaccinated (*n* = 18), of unknown vaccination status (*n* = 11), or were not eligible for vaccination (*n* = 3). Three inpatients (8.3%) had received one MR dose, and one inpatient (2.7%) had received two MR doses.

### 3.3. MeV Genotypes Identified During Outbreaks

**Figure 1:**
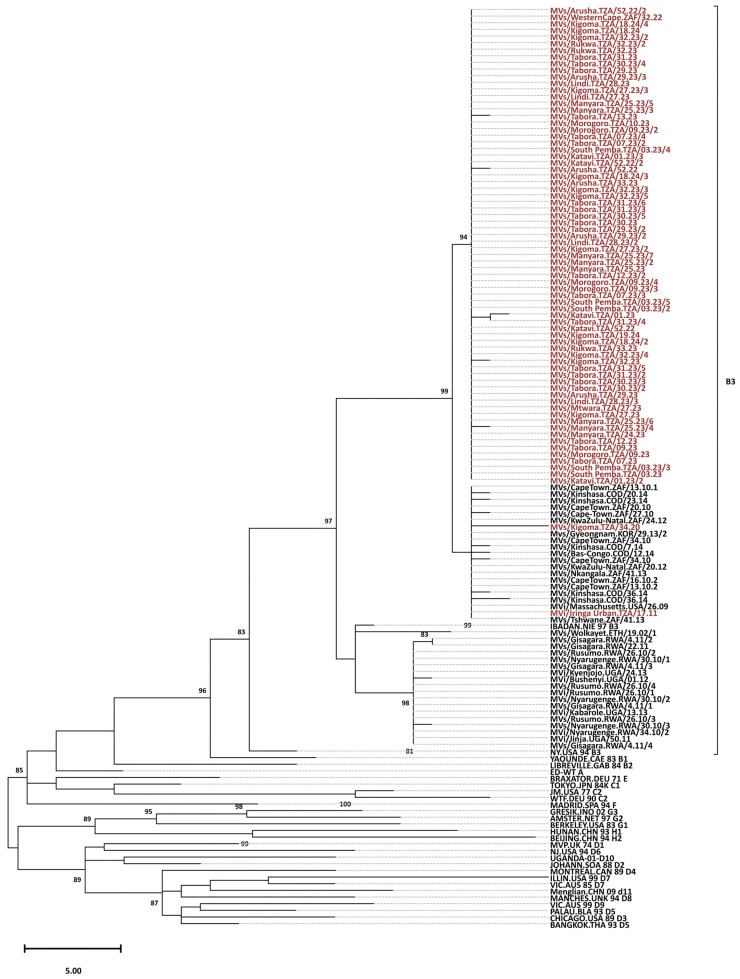
Phylogenetic analysis of the predominant endemic B3 genotype. The tree was constructed based on 450 nucleotides of the C-terminal region of the nucleoprotein gene (N450). The tree was prepared using MEGA and the maximum parsimony model. Bootstrap values are indicated (significant value > 80%). The reference viruses are designated by their GenBank accession numbers. The Tanzanian sequences branching from the 97 nodes are designated by their WHO name (in the MeaNS database). The majority of TZ genotype B3 sequences (red) clustered with other genotype B3 sequences from Africa (black), and 65 were similar with strain GenBank accession number PP987392 from South Africa (Distinct Sequence ID: 8359).

All 73 RT-qPCR positive samples were successfully sequenced and were identified as genotype B3 (Figure 1). All the Tanzania genotype B3 sequences were closely related and clustered with other MeV genotype B3 sequences from Kenya, Ethiopia, Rwanda, Uganda, Burundi, and South Africa. Nine distinct sequence identifiers were detected out of 73 sequences, with the predominance of distinct Sequence ID 8359 that accounted for 65 sequences (89.0%). This ID corresponds to the named strain MVs/Western Cape.ZAF/32.22 with the GenBank accession number PP987392, originally submitted from South Africa to the MeaNS database in August 2022. Other distinct sequence identifiers with one each, included 1310, 8614, 8615, 8616, 8617, 8618, 8619, and 8620. The sample with unique ID 1310 belonged to a named strain MVi/Harare.ZWE/38.09 and was collected during the 2011 measles outbreaks that occurred in Iringa. The remaining seven distinct sequence identifiers did not have named strains in the MeaNS database (Table 3).

### 3.4. Geographical Distribution of Measles Genotypes in Tanzania

**Figure 2:**
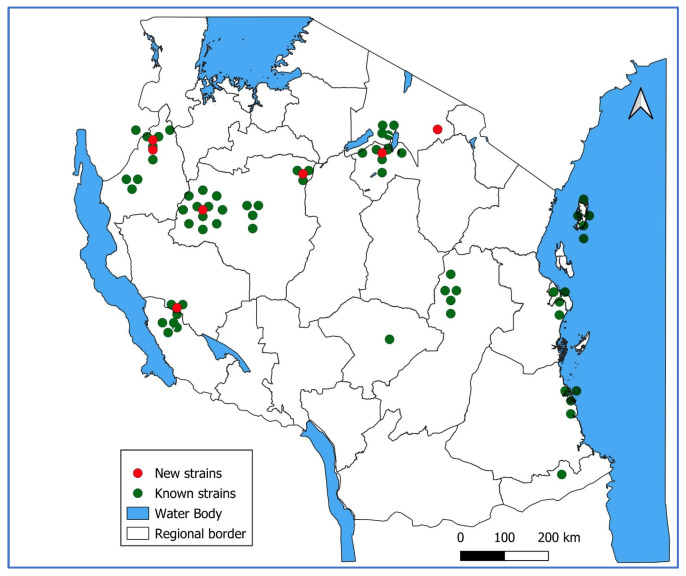
Geographical distribution of genotyped measles cases in Tanzania 2022–2024.

Genotype B3 was obtained from all councils that submitted samples: 6 from Arusha, 4 from Dar es Salaam, 5 from Katavi, 10 from Kigoma, 4 from Lindi, 8 from Manyara, 5 from Morogoro, 01 from Mtwara, 3 from Rukwa, 5 from South Pemba, 01 from Iringa, and 21 from Tabora (Figure 2).

## 4. Discussion

This study has documented the MeV genotype circulating in Tanzania 10 years after introduction of the second dose of the MR vaccine. All 73 strains identified were found to be genotype B3, detected in all 11 regions that submitted samples. Nine unique sequence identifiers were reported among 73 strains, of which seven of them were reported for the first time in Tanzania. The majority of strains, 65 (89.0%), had the unique distinct sequence identifier 8359 for the named MVs/Western Cape.ZAF/32.22, reported for the first time in South Africa.

Out of 129 serum and nasopharyngeal samples collected, 60.4% and 56.4% were measles-specific IgM and RT-qPCR positive, respectively. The observed high positivity could be explained by the timing of sample collection. In this study, serum samples and nasopharyngeal swabs were collected concurrently from cases presenting with fever and rash within 5 days of onset of these symptoms from councils with suspected measles outbreaks. As documented previously, samples collected within time of high viral shedding have been found to have high isolation rates [43].

The inpatient admission status and measles IgM-positive status were significantly associated with RT-qPCR positivity. It was further observed that the majority of those inpatients were either not vaccinated, had unknown vaccination status, or were not eligible for vaccination, indicating an increased risk of being infected with severe disease and requiring admission, as documented previously [44].

The majority of those who were measles IgM positive were also positive on real-time RT-qPCR, with a significant association in the multivariate analysis. Measles IgM antibodies are usually produced in the early stage of the disease, making the presence of measles IgM a strong predictor of viral isolation. Studies have shown that 100% of patients develop IgM antibodies 4 to 11 days after the onset of rash [45].

In this study, the discrepancy between measles IgM positivity and RT-qPCR was observed: 29.4% of those testing measles IgM negative were measles RT-qPCR positive, and 25.6% of those who were measles IgM positive were RT-qPCR negative. This could be explained by immune responses in relation to the concentrations of IgM- and IgG-neutralizing antibodies with viral replications, which can vary between individuals in relation to the timing of sample collection [46]. Xu et al., 2018, reported similar findings, with 14–40% in those with onset of rash within 3 days being RT-qPCR negative despite being measles IgM positive [47]. Further studies to establish the optimal time for sample collection and the dynamics of IgG measles antibodies in relation to RT-qPCR are warranted.

All the samples sequenced in this study belonged to genotype B3, and this genotype was detected in all regions that submitted samples. The MeV genotype B3 has remained endemic in Tanzania for about 19 years, and has persisted despite 10 years of implementing a two-dose MCV policy. These findings are similar to reports from a number of countries in the WHO African Region that have reported the persistence of genotype B3 [9,27,41]. The persistence of genotype B3 could be due to the fact that this genotype is significantly more transmissible than other genotypes, with an R_0_ of 0.64, while the R_0_ for all other genotypes combined is 0.43 [17,48]. The variability in measles transmissibility among genotypes may have important implications for measles control because the vaccination threshold required for elimination may not be the same for all genotypes or among all age groups. Phylogenetic analysis of the MeV genotype B3 from Tanzania and other African countries depicts evidence of a close evolutionary relationship between MeV from Tanzania and strains circulating in other African countries [12]. This is further supported by the fact that 65 strains from Tanzania shared the same unique sequence ID with a strain from South Africa isolated in 2022; named MVs/Western Cape.ZAF/32.22 with the GenBank accession number PP987392 (https://www.ncbi.nlm.nih.gov/nuccore/PP987392) accessed on 22 January 2026.

The genotype data are critical in monitoring the transmission of the virus, especially in this era of measles elimination strategies. It should be noted that the genotype data are useful in assessing the ongoing and constant transmission in countries with endemic measles because, in these countries, many lineages of a single genotype may co-exist as countries begin to move from endemic to epidemic measles. On the other hand, in countries that have eliminated endemic measles, multiple genotypes representing different importation sources without evidence of sustained transmission of any lineage may be detected. In addition, large measles outbreaks caused by a single viral lineage with identical or nearly identical sequences may be detected in countries that had previously high vaccination coverage but have begun to accumulate a larger susceptible population [6,23,30]. Sixty-five (89.0%) of the sequences in this study were identical, confirming large measles outbreaks as a result of the accumulation of a susceptible population.

Despite the fact that the samples genotyped in the current study were not from all regions reporting measles outbreaks, the detected genotype B3 could be responsible for the measles outbreaks that occurred country-wide between 2022 and 2024. This is because only B3 and D8 have been circulating globally since 2020, and D8 is not endemic in Africa. As aspects to note, this study did not perform whole-genome sequencing to further delineate the MeV and establish the evolution of MeV in Tanzania; in addition, the Measles IgG avidity assay was not performed to assess the influence of IgG antibodies in the positivity of RT-qPCR.

## 5. Conclusions

In conclusion, genotype B3 is still endemic in Tanzania and is closely related to other genotype B3 reported globally, indicating its high stability and transmissibility. There is a need to sustain measles genotype surveillance to track circulating wild-type measles strains in the country, and for early detection of the new strains, especially at this time when the country targets measles elimination by 2030 in accordance with AFRO Measles Rubella elimination targets. Identification of seven B3 genotype strains with unique distinct sequence identifiers based on the N450 region shows continuous changes in the MeV, underscoring the need of extended sequencing or WGS to clearly delineate the circulating measles strains in the country, as the standard N450 sequence has limited resolution.

## Figures and Tables

**Table 1:** Sociodemographic and clinical characteristics of 129 suspected measles cases during the 2022–2024 outbreaks in Tanzania.

Variable	*N*	%, Median IQR
** Age in months	129	60: IQR (24–109)
**Age category (years)**		
<5	70	54.3
5–9.99	32	24.8
>10	27	20.9
**Sex**		
Female	58	45.0
Male	71	55.0
**Vaccination**		
Two doses	24	18.6
One dose	13	10.1
Zero dose	52	40.3
Unknown	27	20.9
Not eligible	13	10.1
** Days–onset	129	3: IQR (2–4)
**Admission status**		
Outpatient	93	72.1
Inpatient	36	27.9
**Cough**		
No	20	15.5
Yes	109	84.5
**Running Nose**		
No	36	27.9
Yes	93	72.1
**Joint Pain**		
No	118	91.5
Yes	11	8.5
**Red eyes**		
Yes	48	37.2
No	81	62.8

** Median and interquartile range (IQR).

**Table 2:** Factors associated with measles RT-qPCR positivity among 129 suspected measles cases during the 2022–2024 outbreaks in Tanzania.

Variable		RT-qPCR Results				
		Univariate			Multivariate	
	*N*	Positive (*n*, %)	OR (95% CI)	*p* Value	aOR (95% CI)	*p* Value
** Age in years	129	73, 72: IQR 35–144	1.007 (1.00–1.012)	0.009	1.004 (0.997–1.010)	0.245
**Age category (years)**						
<5	70	36 (51.4)	1			
5–9.99	32	16 (50.0)	0.94 (0.40–2.18)	0.893		
>10	27	21 (77.8)	3.3 (1.19–9.17)	0.022		
**Sex**						
Female	58	29 (50.0)	1			
Male	71	44 (62.0)	1.62 (0.80–3.30)	0.173	1.81 (0.756–4.333)	0.182
**Vaccination**						
Two doses	24	7 (29.2)	1		1	
One dose	13	4 (30.8)	1.08 (0.24–4.69)	0.919	0.877 (0.157–4.875)	0.881
Zero dose	52	35 (67.3)	5.0 (1.74–14.34)	0.003	1.088 (0.287–4.125)	0.900
Unknown	27	21 (77.8)	8.5 (2.4–30.0)	0.001	1.740 (0.373–8.118)	0.481
Not eligible	13	6 (46.2)	2.1 (0.51–8.45)	0.305	0.984 (0.186–5.198)	0.985
** Days–onset	129	73, 3 (IQR: 2–4)	1.04 (0.86–1.25)	0.45		
**Admission status**						
Outpatient	93	45 (48.4)	1		1	
Inpatient	36	28 (77.8)	3.7 (1.5–9.0)	0.004	4.181 (1.33–13.123)	0.014
**Cough**						
No	20	4 (20.0)	1		1	
Yes	109	69 (63.0)	6.9 (2.1–22.1)	0.001	2.438 (0.609–9.755)	0.208
**Running Nose**						
No	36	20 (55.6)	1			
Yes	93	53 (57.0)	1.06 (0.48–2.30)	0.883		
**Joint Pain**						
No	118	65 (55.1)	1			
Yes	11	8 (72.7)	2.17 (0.54–8.60)	0.268		
**Measles IgM**						
Negative	51	15 (29.1)	1			
Positive	78	58 (74.4)	6.96 (3.16–15.31)	<0.001	5.013 (1.726–14.561)	0.003

** Median and interquartile range (IQR) (time from onset of symptoms (rash and fever) to sample collection).

**Table 3:** Descriptions of 7 sequences with new unique sequence identifiers.

SNO	WHO Strain Name	Distinct Seq. Id.	Genotype	Place	Case Onset Date
1	MVs/Arusha.TZA/52.22	8614	B3	Arusha	30 December 2022
2	MVs/Katavi.TZA/01.23	8615	B3	Katavi	1 January 2023
3	MVs/Kigoma.TZA/32.23	8616	B3	Kigoma	10 August 2023
4	MVs/Manyara.TZA/25.23/4	8617	B3	Manyara	20 June 2023
5	MVs/Tabora.TZA/31.23/4	8618	B3	Tabora	3 August 2023
7	MVs/Tabora.TZA/13.23	8619	B3	Tabora	26 March 2023
8	MVs/Kigoma.TZA/34.20	8620	B3	Kigoma	20 August 2020

## Data Availability

All data are included in the manuscript.
